# Supplementary figures and images for: Quantifying wedge-tailed shearwater (Ardenna pacifica) fallout after changes in highway lighting on Southeast Oʻahu, Hawaiʻi
Source: PLoS One. 2022 Mar 24;17(3):e0265832. doi: 10.1371/journal.pone.0265832 (PMC8947263; doi:10.1371/journal.pone.0265832)

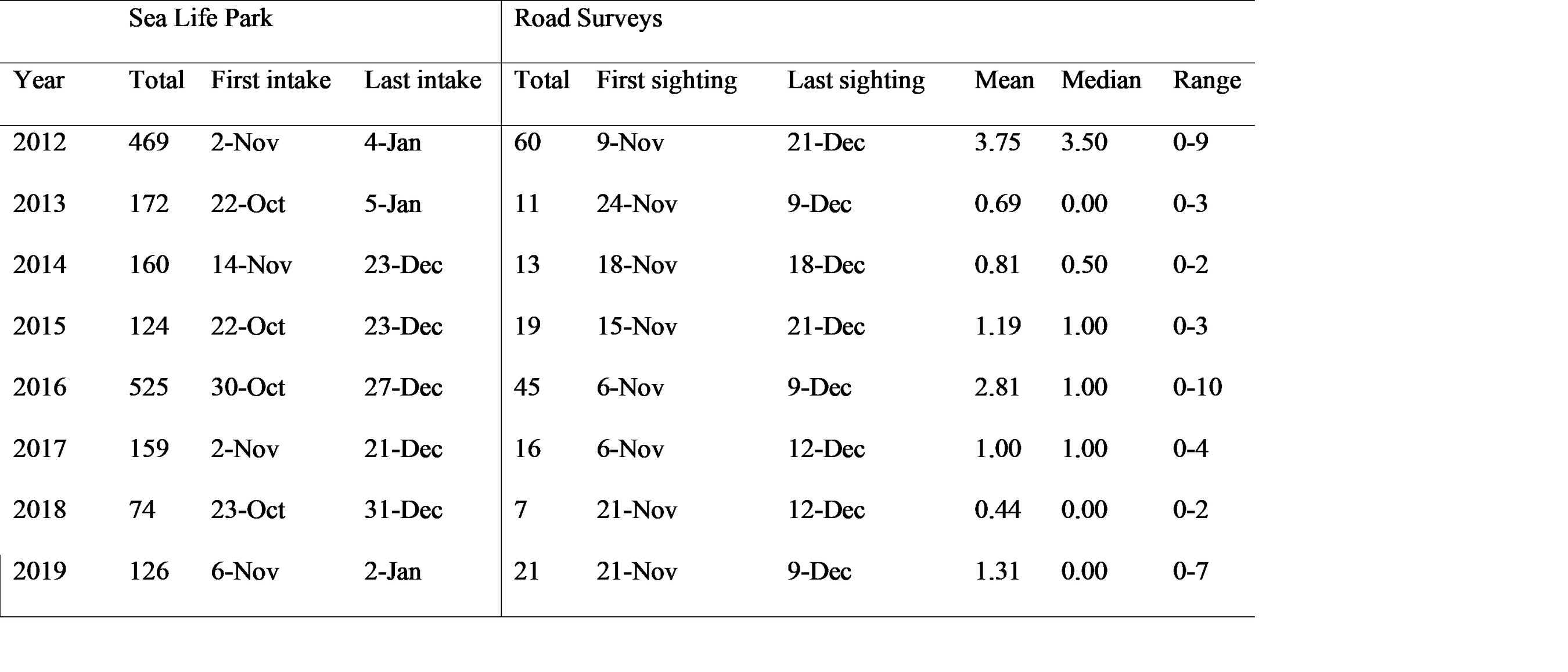

Supplement: S1 Table — Comparison of annual WTSH fallout magnitude (total number of grounded birds) and timing (date ranges) from Sea Life Park intake records and road surveys (this study). Summary statistics (mean, median, and range) refer to the number of grounded birds encountered yearly, based on 16 standardized surveys spanning November 6 to December 21. (TIF) [file pone.0265832.s001.tif]

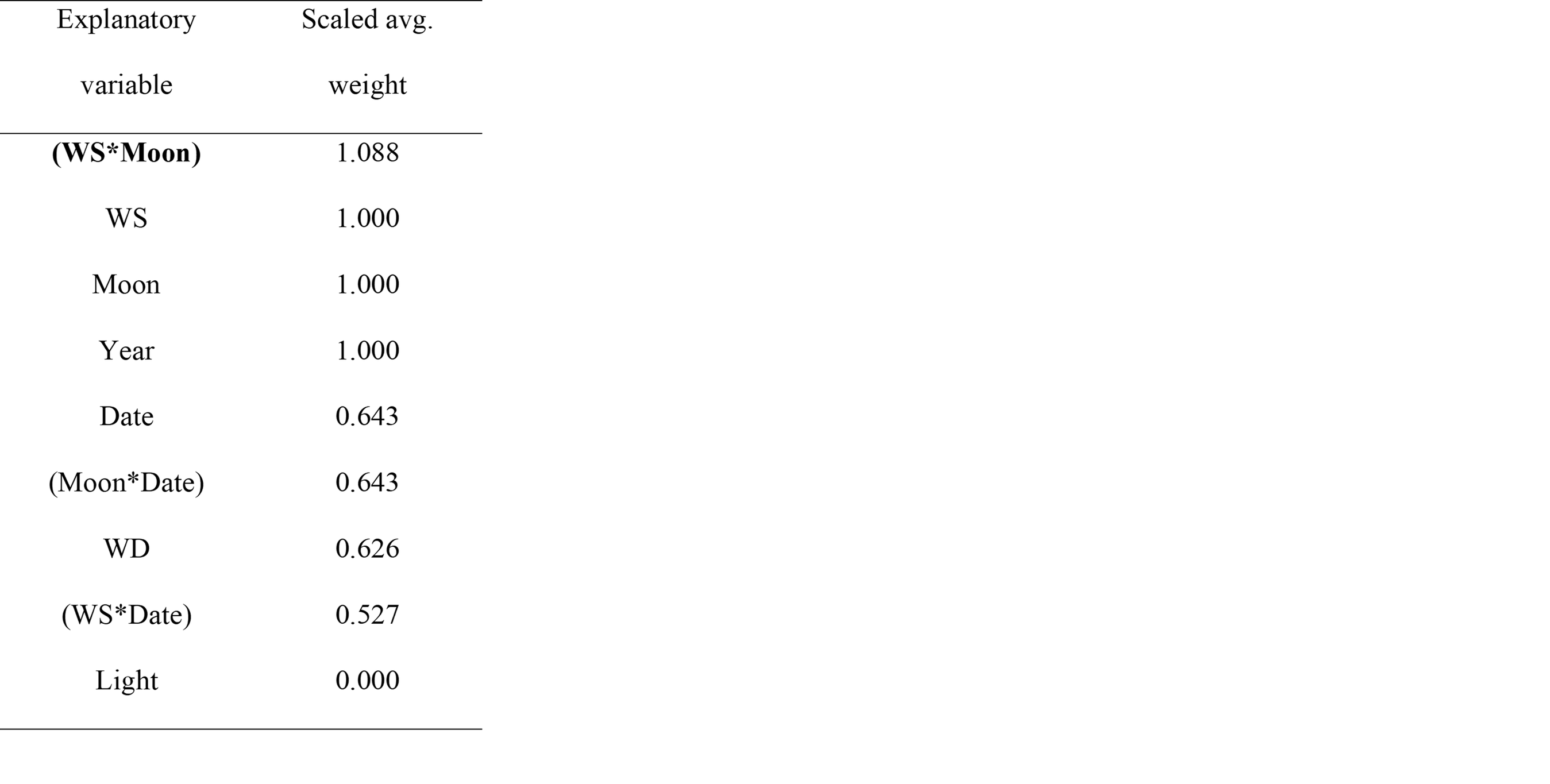

Supplement: S2 Table — Scaled average variable weights. (>1 values indicate greater than average weight when variable was included in model; weights = 1 are average, weights <1 less than average). (TIF) [file pone.0265832.s002.tif]

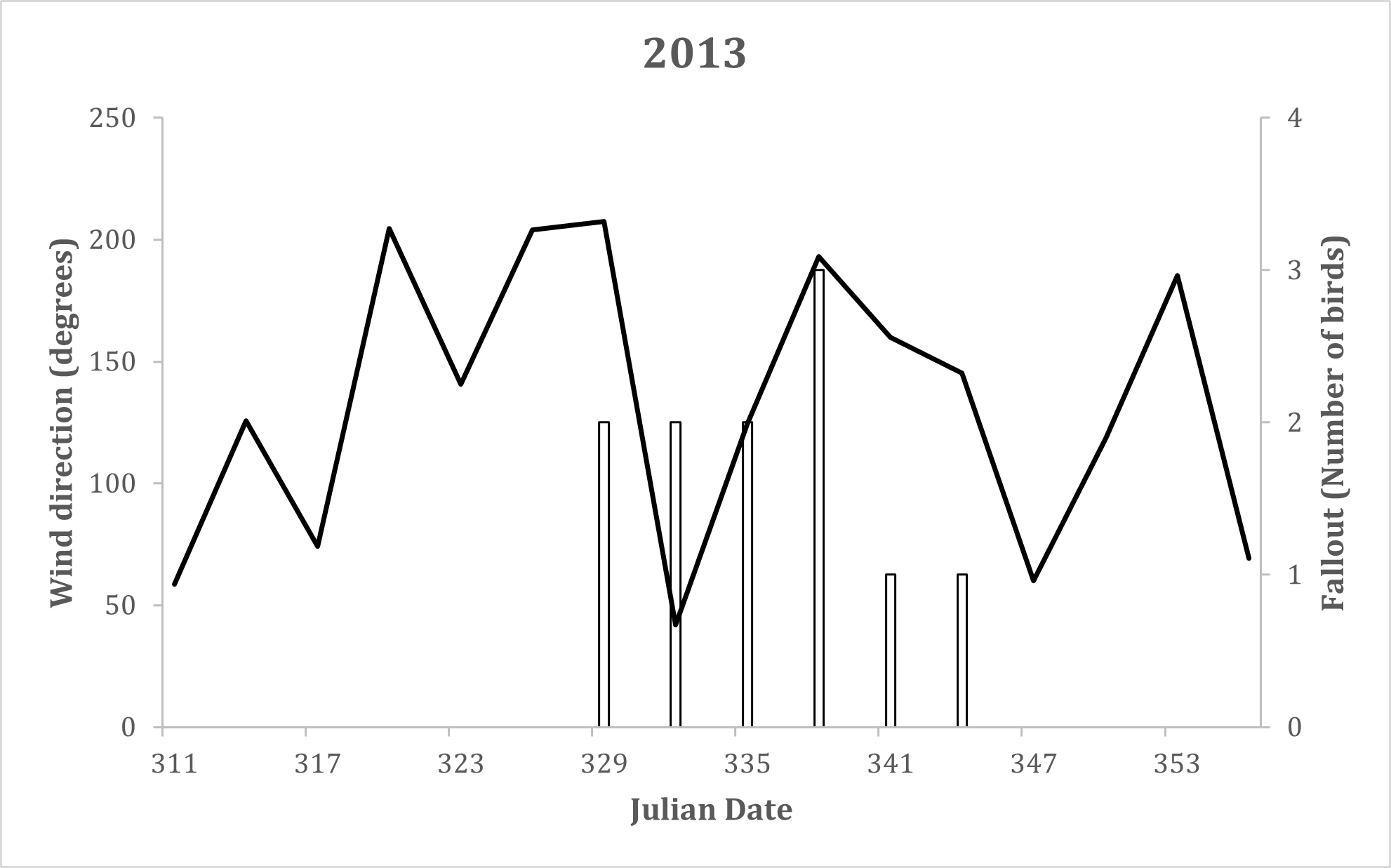

Supplement: S1 Fig — Wind direction and fallout during the 2013 fledging season. Black line is wind direction and white bars are number of birds per survey. (TIF) [file pone.0265832.s003.tif]

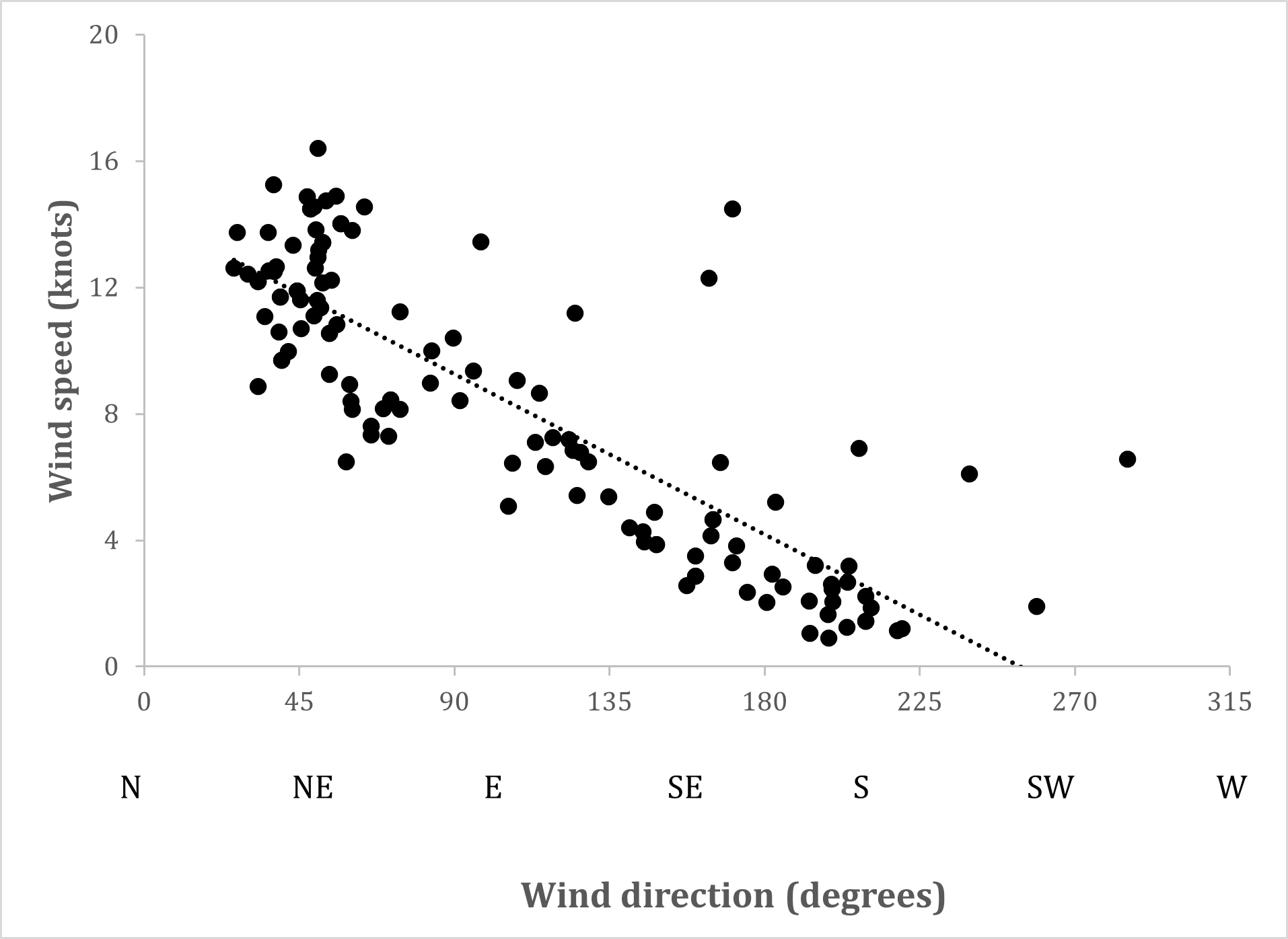

Supplement: S2 Fig — Scatterplot of wind speed and wind direction during the fledging seasons 2012–2019 (R2 = 0.71). (TIF) [file pone.0265832.s004.tif]
