# Supplementary material for: Quantifying wedge-tailed shearwater (Ardenna pacifica) fallout after changes in highway lighting on Southeast Oʻahu, Hawaiʻi
Source: PLoS One. 2022 Mar 24;17(3):e0265832. doi: 10.1371/journal.pone.0265832 (PMC8947263; doi:10.1371/journal.pone.0265832)
Supplement: Attachment — Submitted filename: Review Urmston et al.docx [file pone.0265832.s006.docx]

This manuscript reveals a novel analysis of the effect of moon, wind speed and wind direction on the fallout of wedge-tailed shearwaters. Additionally, there was a transition from high-pressure sodium lamps to LED lamps on the route of the regular fallout survey. This provided a unique opportunity to study the effect of the change in streetlamps on the number of grounded birds. The study reveals that while the transition from HPS to LED streetlamps did not influence the fallout, the interaction between moon and wind speed did. While the data and analysis provide a new insight into the grounding of burrow-nesting shearwaters, I believe that the manuscript itself could be improved. Firstly, I think that moving sections of full model before the yearly models could improve the focus of the study. Furthermore, the authors could consider including the timing of moonrise, moonset, as well as cloud cover in their model. Finally, I include minor points that could improve clarity and readability of the manuscript. My comments include first words of each line I referred to, since no line numbers were provided.

ABSTRACT

1. ‘Fallout’, ‘wedge-tailed shearwater’ and ‘Hawaii’ is already in the title, I propose to use a different keyword (e.g. grounding, ALAN, artificial light, Procellariiform).
2. “for the first time. In Hawaiʻi, fledgling”

Second sentence of the abstract suggests that wedge-tailed shearwater must collide with an obstacle to become grounded. Other shearwaters usually ground without first encountering an obstacle. Thus, I would re-word this sentence if it is the same for wedge-tailed shearwater.

3. “Our main goal was to compare”

It sounds counterintuitive that your main goal was to check the effect of transition between streetlights, but this is not mentioned in the title. I think the focus on both, the transition, and environmental variables is fair, but here I would indicate that it was “one of your goals” or simply write “our aim…”.

4. “of a fallout response to the change from”

I would be careful with recommending changes to energy saving LED lights throughout the Hawaiian archipelago in the last sentence of the abstract. LED can come in many different intensities, spectra and spread. In here you specifically checked if transition from HPS to shielded 3000K and 4000K LED affected the fallout. Thus, I would still recommend caution when using LEDs.

INTRODUCTION

1. I propose adding two references to statement “coastal light pollution can disrupt adult seabirds provisioning their chicks on colonies (Telfer, et al. 1987)”.

Cianchetti-Benedetti, et al. 2018. Conflicts between touristic recreational activities and breeding shearwaters: short-term effect of artificial light and sound
on chick weight. https://doi.org/10.1007/s10344-018-1178-x

Syposz M, Padget O, Willis J, Doren BMV, Gillies N, Fayet AL, et al. Avoidance of different durations, colours and intensities of artificial light by adult seabirds. Scientific Reports. 2021;11: 18941. doi:10.1038/s41598-021-97986-x

2. Repetition: “the prevailing environmental and celestial conditions (Ainley et al., 2001; Telfer et al., 1987) **~~conditions~~**”
3. “nests are attracted to onshore lighting…”

I would recommend refrain from using word ‘attracted’ and replace it with ‘affected’ here and throughout the manuscript. We do not really know why burrow-nesting seabirds are affected by lights and attraction is just one of the hypotheses.

4. “1987) conditions, and the attractiveness of anthropogenic…”

I would avoid the word “attractiveness” as we do not really know if birds are attracted or disoriented by the light. I would suggest using “and the **features** of anthropogenic lights”.

5. “Birds are attracted to bright light sources including streetlights, sports fields and urbanized areas…” . But also, vessels e.g.:

JP Glass & PG Ryan (2013) Reduced seabird night strikes and mortality in the Tristan rock lobster fishery, African Journal of Marine Science, 35:4, 589-592. http://dx.doi.org/10.2989/1814232X.2013.860049

6. “on the visual perception of wedge-tailed shearwaters (Ardenna pacifica – previously Puffinus …”

Remove italics (*Ardenna pacifica –* **previously** *Puffinus pacificus*)

7. Unnecessary repetition, I would recommend removing (November – December) from sentace: “Starting in 2002, USFWS initiated a program of opportunistic road surveys of the southeast section of Oʻahu during the fledging season **~~(November – December)~~** …”
8. “Friswold et al. (2020) documented an increasing trend in annual fallout numbers between 2003 and 2010, and a two- year cycle of alternating years of high and low fallout. These results are suggestive of the potential influence of the breeding population size and the annual reproductive success on fallout.”

I wonder if increasing trend in annual fallout couldn’t be indicative of more light features fixed in Hawaii. Furthermore, two-year cycle could actually indicate that the moon has an important effect on grounding of shearwaters. It has been previously reported that full moon coinciding with peak fledging date can diminish the fallout (Rodriguez et al. 2014). Thus, I suggest using a different study to indicate that reproductive success and population size has an effect:

Ainley, D.G., Podolsky, R., Deforest, L. & Spencer, G. 2001. The Status and Population Trends of the Newell’s Shearwater on Kaua’i: Insights from Modeling. *Stud. Avian Biol.* 22: 108–123.

9. “Yet, to date, only one study has investigated the environmental drivers of WTSH fallout, by comparing a “wreck” year of unusually high fallout (1994), when WTSH groundings increased ten-fold from the two “normal” years prior.”

This sentence needs citation.

10. “yellow-orange HPS lights to shielded 3000 – 4000 K white LED lights.”

It would be beneficial here to explain Kelvins here, as it is not an intuitive measure of light.

11. “by repeatedly surveying a fallout hostpot in the natural course of the fledging season.”

I would consider re-writing the end of this sentence to “repeatedly surveying a fallout hostpot during the fledging season”.

12. “and strong northeasterly trade winds (>8 mph).”

Could you explain briefly why you decided that low moon, strong and northeasterly wind will cause a higher fallout?

METHODS

1. “We related WTSH fallout to publicly-available environmental datasets…”

It would be interesting to know where and how far the weather station was.

2. “U.S. Naval Observatory (<http://aa.usno.navy.mil/data/docs/MoonFraction.php)>.”

For some reason the link does not work.

3. “the average wind speed (mph), wind direction (degrees), and lunar disk illumination (%) from the three…”

I understand that you recalculated the wind speed from knots to mph. It would be good to add that information.

4. “We fitted nine models: eight separate yearly models explored interannual variation in”

Shouldn’t be intra-annual variation?

5. “…is more appropriate (Ver Hoef and Boveng, 2007).”

I would suggest explaining here that the negative binomial distribution was used. As it is not clear from the text up until later that there were a few instances where negative binomial distribution was used.

6. “wind speed (knots), average wind direction (degrees), and Julian date (the number of days since”

It is unclear if you used wind speed in knots or mph. Please, be consistent throughout the text.

7. “We used the AICmodavg package (R Core Team 2019) to conduct a model fit assessment with…”

I suggest citing

“Mazerolle, M. J. *AICcmodavg: Model selection and Multimodel Inference based on (Q)AIC(c)* R package version 1.31,

<http://cran.r-project.org/web/packages/AICcmodavg/index.html> (2013)”

RESULTS

1. “18.7 S.D. (median = 17.5) (Table A1). There was a strong correlation…”

This correlation was not mentioned in the methods section. Please, include a description in method section.

2. “Two years (2012 and 2016) showed significantly higher…”

The fact that these two years are significantly higher is repeated in the next section *Full Model*. Since we know that these years are significantly higher thanks to the multi-year model, I recommend you re-phrase this sentence.

3. “(Figure 6). In 2016, a single new moon…”

I think Figure 6 appeared here by mistake. Figure 4 and Figure 6 in next sentence should be replaced by Figure 5 at the end of the sentence.

4. I think, we do not have figure 4 described on page 15.
5. I think it would be good to further clarify the description of Table 2 and include the distribution used in this model. Perhaps something like: “Results of best-fit model that included numbers of carcasses from all the years of the road survey. The reference year (intercept) is year 2012.”

DISCUSSION

General point: I would suggest restructuring the discussion. The discussion starts from deliberation about yearly differences and patterns found there, while the full model was the source of the main findings and goal of this paper, namely that interaction between moon and wind influences the fallout and that transition in streetlights did not. Reader must come to almost the end of the discussion to read about those findings. I would also consider congregate limitations in one paragraph, as they are affecting the results of full and yearly models.

1. “The strong correlation between the yearly numbers of grounded WTSH…”

It is a subject of discussion whether a regular survey could be reflection of citizen-science efforts or rather it should be the other way round. Citizen-science efforts reflect patterns found through regular surveying. What I mean here is that if I had just citizen-science data, I would argue that they would not be able to properly reflect larger-scale fallout trends due to variable effort from citizens. Since, however, they are highly correlated with regular survey, they are more likely to reflect the large-scale pattern. I would re-word this first sentence of your discussion to make your point stronger and clearer.

2. “fledging. However, if most birds fledge within an hour after sunset,”

Indeed, most of the groundings occur soon after the sunset, see Reed 1985 and

Rodríguez, A., García, D., Rodríguez, B., Cardona, E., Parpal, L. & Pons, P. 2015. Artificial lights and seabirds: Is light pollution a threat for the threatened Balearic petrels? *J. Ornithol.* 156: 893–902.

3. “clouds obscure a rising moon from a given natal colony, then navigational benefits provided by…”

I wonder why authors did not decide to use the cloud cover as one of the variables. It would be good to clarify if this was not available.

Furthermore, I believe that information on the height of moon above horizon are freely available with package ‘oce’ in R, the function is called moonAngle(). Perhaps, using this parameter would allow to further disentangle the effect of moon. For example, by multiplying the percentage of time above horizon between hours 18:00-6:00 and the visible fraction of the moon.

There is another way of calculating moon illuminance that takes into account a number of astronomical factors described in Rubolini et al. 2015. It also includes the time of moonset and moonrise, but it uses Fortran software. I would be keen to get those data for you if you struggle.

“Rubolini, D., Maggini, I., Ambrosini, R., Imperio, S., Paiva, V.H., Gaibani, G., Saino, N. & Cecere, J.G. 2015. The Effect of Moonlight on Scopoli’s Shearwater Calonectris diomedea Colony Attendance Patterns and Nocturnal Foraging: A Test of the Foraging Efficiency Hypothesis. *Ethology* 121: 284–299.”

4. “The peak fledging times of WTSH are unknown but could be useful”

I think the limitation of your results that effect not only the influence of the moon, but also all the other variables should be stated later. Furthermore, it would be good to mention that there is no information on fluctuation in population size, as well as yearly breeding success that would also affect the fallout.

5. “possible explanation for why we observed more fallout

This sentence suggest that strong winds do not enable WTSH fledging. Please, re-word this sentence. I would suggest adding information about necessary phase where young birds need to gain experience and develop muscle reinforcement.

de Grissac, S., Bartumeus, F., Cox, S. L. and Weimerskirch, H. 2017. Early-life foraging: behavioral responses of newly fledged albatrosses to environmental conditions. – Ecol. Evol. 7: 6766–6778.

Fayet, A. L. 2020. Exploration and refinement of migratory routes in long-lived birds. – J. Anim. Ecol. 89: 16–19.

6. “toward land and, thus into our study area, where they are attracted…”

Again, I would refrain from using word ‘attracted’ and use word ‘affected’. If all the birds were attracted, there should have been much higher number of them grounding since I presume, they are able to see the lights from their colony. Or do you perhaps suggest that there is a threshold at which birds are attracted towards the light and only when they are closer to it, buffeted by wind, they show attraction. Why not disoriented?

7. “direction, an exception to this general pattern was observed in 2013, when peak fledging”

I understood that you do not know the peak fledging date. I guess, you meant the peak fallout dates. Please, clarify.

8. “windward coast of the island (Work and Rameyer, 1999).

Could it be that birds in that study were from different colony? Does it mean that the hotspot that year was in South of the island?

9. “windward coast of the island (Work and Rameyer, 1999). The lack of significance of wind”

I would specify here “The lack of significance of wind direction in other years than 2013…”

10. “phase was closer to a new moon on that date. It appears that when peak fledging coincides with a…”

Please, clarify if you have data on peak fledging date. If yes, please use them in the model.

11. “significant (R2=0.038, F2,5=1.140, p=0.39),”

I think it would be beneficial to have details of the methodology of this analysis in supplementary materials. I wonder why you would expect quadratic relationship.

12. “(2019) fallout, is inherently difficult to model.”

Why are they inherently difficult to model? As mentioned before, I think since full model should get ‘highest consideration’ than it should come first in the discussion. I further would suggest removing this paragraph as it is repetitive.

13. “of these variables, captured using their interactions with date, were less important.”

Less important or non-significant?

14. “lighting adjustments that will mitigate fallout, our study shows that the change to the shielded”

I like this recommendation. Perhaps, just specify that they were 3000K and 4000K LEDs.

15. “variety of CCTs with options as low as 2200 o K”

Please, choose either “o K” or “K” sign and be consistent throughout the manuscript.

16. “A recent survey of lighting experts suggests that while LEDs…”

This paragraph finishes quite abruptly, and it seems to fit better into introduction. Or perhaps a sentence of recommendation of future studies using different types of LEDs in areas where seabirds ground would smooth the transition.

17. “Our results are encouraging because they suggest that the shielded”

In what sense encouraging?

18. “colonies could be targeted for localized management.”

Add reference to Friswold et al. 2020.

REFRENCES

1. Rodríguez et al., 2017 (Seabird mortality induced by land-based artificial lights) lacks letter “a”.

FIGURES

1. You mentioned streetlights illuminating highway, which I presume are next to the survey road, and inland lights. I wondered if it would be possible to circle the area where the lights were changed in Figure 1, for clarification.
2. I suggest including more descriptive axis captions in Figure 2 i.e. “Number of WTSH rescued by Sea Life Park”, “Number of carcasses found during road surveys”.
3. I suggest including a legend explaining the meaning of the radius of the circle in Figure 4.
4. I suggest adding moon illumination to Figure 5. It could be added as a dashed line, or the days of full moon could be shaded vertically on the graph.
