# Supplementary material for: Quantifying wedge-tailed shearwater (Ardenna pacifica) fallout after changes in highway lighting on Southeast Oʻahu, Hawaiʻi
Source: PLoS One. 2022 Mar 24;17(3):e0265832. doi: 10.1371/journal.pone.0265832 (PMC8947263; doi:10.1371/journal.pone.0265832)
Supplement: Attachment — Submitted filename: PONE-D-21-31078_R1_ResponseReviewers_122821.docx [file pone.0265832.s007.docx]

Re: Response to Reviewers (PONE-D-21-31078)	12/28/2021

Dear Dr. Vitor Hugo Rodrigues Paiva,

Thank you for giving us the opportunity to submit a revised draft of our manuscript previously entitled “Timing and magnitude of Wedge-tailed Shearwater (*Ardenna pacifica)* fallout on Southeast Oahu, Hawaii”, now titled “Quantifying wedge-tailed shearwater (*Ardenna pacifica*) fallout after changes in highway lighting on Southeast Oʻahu, Hawaiʻi”. We sincerely appreciate the time and effort that you and the reviewers have dedicated to reviewing and improving our manuscript. We are also grateful to the reviewers for their insightful comments and edits. We have incorporated all the changes requested by the reviewers, except adding the analysis of the number of rescued shearwaters, to augment the current analyses of the number of grounded shearwaters detected during road surveys due to the inherent spatial and temporal mis-matches of the two datasets, which inhibit a side-by-side modelling analysis. Moreover, we were concerned that doubling the number of models we discuss would greatly lengthen the paper and deter from the main results, by forcing to discuss how the various models agreed or disagreed. We explain our rationale in great detail in the response to reviewers section.

We are forwarding two versions of the revised (R1) ms: (i) a “marked-up” version includes all the edits we implemented using track changes in Microsoft Word, and (ii) a “clean” version after accepting all these changes and edits. Below, we also provide a point-by-point response to the reviewers’ comments and concerns.

Finally, we provide an amended Role of Funder statement, to reflect that this work was supported by Experiment.com (Blinded by the light: reducing shearwater deaths along a coastal highway in O 'ahu, Hawai'i) and The Eppley Foundation for Research (Blinded by the Light: Shearwater Deaths Along a Coastal Highway in O'ahu). The funders had no role in study design, data collection and analysis, decision to publish, or preparation of the manuscript.

We look forward to your decision.

Sincerely,

Jennifer Urmston, Keith Swindle and David Hyrenbach

**RESPONSES TO REVIEWER COMMENTS**

**Journal Requirements**

**Comment 1:** *Please ensure that your manuscript meets PLOS ONE's style requirements, including those for file naming. The PLOS ONE style templates can be found at*

[*https://journals.plos.org/plosone/s/file?id=wjVg/PLOSOne_formatting_sample_main_body.pdf*](https://journals.plos.org/plosone/s/file?id=wjVg/PLOSOne_formatting_sample_main_body.pdf) *and*

[*https://journals.plos.org/plosone/s/file?id=ba62/PLOSOne_formatting_sample_title_authors_affiliations.pdf*](https://journals.plos.org/plosone/s/file?id=ba62/PLOSOne_formatting_sample_title_authors_affiliations.pdf)

**Response:** We have formatted the ms according to the journal requirements.

**Comment 2:** *Thank you for stating the following financial disclosure:*

*“This work was supported by Experiment.com (Blinded by the light: reducing shearwater deaths along a coastal highway in O 'ahu, Hawai'i) and The Eppley Foundation for Research (Blinded by the Light: Shearwater Deaths Along a Coastal Highway in O'ahu)”*

*Please state what role the funders took in the study.  If the funders had no role, please state: "The funders had no role in study design, data collection and analysis, decision to publish, or preparation of the manuscript."*

*If this statement is not correct you must amend it as needed.*

*Please include this amended Role of Funder statement in your cover letter; we will change the online submission form on your behalf.*

**Response:** We provided an amended Role of Funder statement in our cover letter, to reflect that this work was supported by Experiment.com (Blinded by the light: reducing shearwater deaths along a coastal highway in O 'ahu, Hawai'i) and The Eppley Foundation for Research (Blinded by the Light: Shearwater Deaths Along a Coastal Highway in O'ahu). The funders had no role in study design, data collection and analysis, decision to publish, or preparation of the manuscript.

**Comment 3:** *In your Data Availability statement, you have not specified where the minimal data set underlying the results described in your manuscript can be found. PLOS defines a study's minimal data set as the underlying data used to reach the conclusions drawn in the manuscript and any additional data required to replicate the reported study findings in their entirety. All PLOS journals require that the minimal data set be made fully available. For more information about our data policy, please see* [*http://journals.plos.org/plosone/s/data-availability*](http://journals.plos.org/plosone/s/data-availability)*.*

*Upon re-submitting your revised manuscript, please upload your study’s minimal underlying data set as either Supporting Information files or to a stable, public repository and include the relevant URLs, DOIs, or accession numbers within your revised cover letter. For a list of acceptable repositories, please see* [*http://journals.plos.org/plosone/s/data-availability#loc-recommended-repositories*](http://journals.plos.org/plosone/s/data-availability#loc-recommended-repositories)*. Any potentially identifying patient information must be fully anonymized.*

*Important: If there are ethical or legal restrictions to sharing your data publicly, please explain these restrictions in detail. Please see our guidelines for more information on what we consider unacceptable restrictions to publicly sharing data:* [*http://journals.plos.org/plosone/s/data-availability#loc-unacceptable-data-access-restrictions*](http://journals.plos.org/plosone/s/data-availability#loc-unacceptable-data-access-restrictions)*. Note that it is not acceptable for the authors to be the sole named individuals responsible for ensuring data access.*

*We will update your Data Availability statement to reflect the information you provide in your cover letter.*

**Response:** We provided a data file for inclusion as supplementary table 2, which includes all road survey observations and environmental data, sampled every three days between Nov. 6 and Dec. 21 for eight years (2012 – 2019). These were the data used to develop the multi-year full model and the eight single-year yearly models.

**Comment 4:** *We note that Figure 1 in your submission contain map images which may be copyrighted. All PLOS content is published under the Creative Commons Attribution License (CC BY 4.0), which means that the manuscript, images, and Supporting Information files will be freely available online, and any third party is permitted to access, download, copy, distribute, and use these materials in any way, even commercially, with proper attribution. For these reasons, we cannot publish previously copyrighted maps or satellite images created using proprietary data, such as Google software (Google Maps, Street View, and Earth). For more information, see our copyright guidelines:* [*http://journals.plos.org/plosone/s/licenses-and-copyright*](http://journals.plos.org/plosone/s/licenses-and-copyright)*.*

*We require you to either (1) present written permission from the copyright holder to publish these figures specifically under the CC BY 4.0 license, or (2) remove the figures from your submission:*

*a. You may seek permission from the original copyright holder of Figure 1 to publish the content specifically under the CC BY 4.0 license.*

*We recommend that you contact the original copyright holder with the Content Permission Form (*[*http://journals.plos.org/plosone/s/file?id=7c09/content-permission-form.pdf*](http://journals.plos.org/plosone/s/file?id=7c09/content-permission-form.pdf)*) and the following text:*

*“I request permission for the open-access journal PLOS ONE to publish XXX under the Creative Commons Attribution License (CCAL) CC BY 4.0 (*[*http://creativecommons.org/licenses/by/4.0/*](http://creativecommons.org/licenses/by/4.0/)*). Please be aware that this license allows unrestricted use and distribution, even commercially, by third parties. Please reply and provide explicit written permission to publish XXX under a CC BY license and complete the attached form.”*

*Please upload the completed Content Permission Form or other proof of granted permissions as an "Other" file with your submission.*

*In the figure caption of the copyrighted figure, please include the following text: “Reprinted from [ref] under a CC BY license, with permission from [name of publisher], original copyright [original copyright year].”*

*b. If you are unable to obtain permission from the original copyright holder to publish these figures under the CC BY 4.0 license or if the copyright holder’s requirements are incompatible with the CC BY 4.0 license, please either i) remove the figure or ii) supply a replacement figure that complies with the CC BY 4.0 license. Please check copyright information on all replacement figures and update the figure caption with source information. If applicable, please specify in the figure caption text when a figure is similar but not identical to the original image and is therefore for illustrative purposes only.*

*The following resources for replacing copyrighted map figures may be helpful:*

*USGS National Map Viewer (public domain):* [*http://viewer.nationalmap.gov/viewer/*](http://viewer.nationalmap.gov/viewer/)

*The Gateway to Astronaut Photography of Earth (public domain):* [*http://eol.jsc.nasa.gov/sseop/clickmap/*](http://eol.jsc.nasa.gov/sseop/clickmap/)

*Maps at the CIA (public domain):* [*https://www.cia.gov/library/publications/the-world-factbook/index.html*](https://www.cia.gov/library/publications/the-world-factbook/index.html) *and* [*https://www.cia.gov/library/publications/cia-maps-publications/index.html*](https://www.cia.gov/library/publications/cia-maps-publications/index.html)

*NASA Earth Observatory (public domain):* [*http://earthobservatory.nasa.gov/*](http://earthobservatory.nasa.gov/)

*Landsat:* [*http://landsat.visibleearth.nasa.gov/*](http://landsat.visibleearth.nasa.gov/)

*USGS EROS (Earth Resources Observatory and Science (EROS) Center) (public domain):* [*http://eros.usgs.gov/#*](http://eros.usgs.gov/)

*Natural Earth (public domain):* [*http://www.naturalearthdata.com/*](http://www.naturalearthdata.com/)

**Response:** Figure 1 has not been previously published elsewhere but is an original created using Arc GIS Pro. This figure shows a series of geographic features overlaid on the ArcGIS Pro Software Version 2.5 topographic basemap (ESRI 2020). To this end, we added a citation to the references section:

ESRI (2020). ArcGIS Pro (Version 2.5). Esri Inc. https://www.esri.com/en-us/arcgis/products/arcgis-pro/overview.

**Comment 5:**  *Please include a copy of Table A1 which you refer to in your text on page 11.*

**Response:** We apologize for this oversight. We added this table, relabeled as Table S1, in the Supplementary materials at end of manuscript.

**Comment 6:** *Please upload a copy of Supporting Information Figure S1 and S2 which you refer to in your text on page 18 and 19.*

**Response:** We apologize for this oversight. We have uploaded Figures S1 and S2, in the Supplementary materials at end of manuscript.

**Comments from Reviewer 1**

**Comment 1:** *Firstly, I think that moving sections of full model before the yearly models could improve the focus of the study.*

**Response:** Thank you for the suggestion. We reorganized the ms and now focus on the multi-year model, which addresses the shift from the HPS to the LED lights. Then, we discuss the year-to-year variability using the single-year models.

**Comment 2:** *Furthermore, the authors could consider including the timing of moonrise, moonset, as well as cloud cover*

**Response:** While we agree that developing a more comprehensive metric if lunar illumination that includes the timing of moonrise / moonset and the cloud cover could improve the fit of the model, we chose to not do so for two reasons: (1) complexity of weather conditions and (2) applicability of the model results. Thus, we respectfully would prefer to not modify the current environmental datasets and resulting models.

It is inherently difficult to obtain a cloud cover metric that accurately describes our study site, which encompasses a section of the windward and a section of the leeward coasts of our island. Because storms sweep across from the north or from the south, the weather on the windward and leeward shores of our island is often different. Thus, cloudiness and precipitation are often very different on either side (northwest or southeast) of Makapuu Point. Thus, developing a metric for the entire survey would require a weighing the average of the cloud cover on both shores, using the relative survey length on along shores. While this approach is doable, we fear that it would hinder the applicability of our model results, for resource managers and wildlife rescue organizations.

Thus, to ensure the wide use of our model results, we decided to used “simple” environmental variables available from internet datasets: percent moon illumination from the US Naval Observatory and hight-time (6 pm to 6 am) hourly wind speed from the Pacific Islands Ocean Observing System. Using these variables will allow managers and wildlife rescuers to develop predictive models using wind data predictions and the lunar almanac.

**Comment 3:** *Finally, I include minor points in the attachment that could improve clarity and readability of the manuscript*

**Accepted most minor edits and made changes accordingly in manuscript.**

**Comments from Reviewer 2**

**Comment 1***: I found the methods section too detailed and would suggest compressing some information and maybe place it in a supplement material annex*

**Response:** We reorganized the methods section and moved some of the material to a “Fallout Modelling” section, to minimize redundancy and streamline the methodological descriptions. While we did not manage to shorten this section substantially, we feel this information is useful so readers can fully evaluate the data analysis. Thus, we would like to retain this material in the ms, rather than moving it to the supplementary materials section.

**Comment 2:** *Both the results and the discussion highlight the environmental aspects of the analysis while the LED is nearly absent from the results and, albeit well discussed, does not have a prominent placement in the discussion. I would suggest rearranging the paper in a way that highlights the LED vs HPS change as this is a most crucial information for current light pollution mitigation efforts.*

**Response:** Originally, we focused the ms on the environmental drivers of fallout, rather than on the influence of the changes in highway lighting, because the statistics highlighted the significant influences of moonlight and wind and did not find an overall difference before / after the changes in lighting regimes. Yet, given the reviewer’s suggestions we have refocused the ms to highlight the analysis of the lighting regimes in two ways: (i) we have modified the title of the ms, and (ii) we have rearranged the material in the methods / results / discussion sections, by placing the multi-year model before we discuss the yearly models.

**Comment 3:** *I would also suggest that the authors increase the analysis of the data by including if possible the total fallout per year registered by the SLP which correspond to the survey area, thus increasing the scope of light regime change analysis. You provide the totals in Fig.2 it would be possible to add this data to the light regime comparison? I understand that by drawing a parallel between SLP fallout records and your survey dead birds you are showing that your data is potentially indicative of the overall fallout and I agree, however I feel the analysis and results feel flat and could be worked on to present a more robust evidence.*

**Response:** While analyzing the SLP intake fallout records would be a valuable and insightful exercise, we feel that this analysis is not appropriate in our current ms for three reasons:

First, there is a spatial mis-match of the two datasets: our surveys focus on a fallout hotspot within a relatively small geographic area, and the SLP intake records include island-wide returns of grounded birds. Moreover, because most of the grounded birds do not include detailed location information, usually merely the city or highway where they were found, it would be impossible for us to subset these records to only analyze returns from our survey area. In fact, 20 – 40 of the records in any given year, do not include any location data. Moreover, while we know when the lighting regime changes for our survey area occurred between spring and summer of 2016, the shift from HPS to LEDs has been a lengthy process, with different areas of the island transitioning at different times over the span of a 2-year period (2015 – 2017). Thus, we would not be able to break up the SLP time series into a HPS vs an LED lighting regime. These uncertainties would greatly complicate the analysis of fallout trends.

Second, there is an environmental driver mismatch: due to the different spatial extent and the timing (daily vs every 3 days) of the two datasets, we would have to either perform the SLP analysis using daily environmental data or we would have to aggregate the SLP data into 3-days periods to match the timing of the road surveys. The former approach would complicate the interpretation of the two separate analyses, and the latter approach would miss the fine temporal resolution in the SLP data.

Finally, due to these inherent mis-matches, we are concerned that including both analyses in this ms would greatly blur the main messages of the ms, because we would be discussing and comparing four analyses: two multi-year models (SLP intake and road surveys) and two sets of yearly models (SLP intake and road surveys). If the model results agreed, it would still be difficult for the readers to digest all of these results. If the model did not agree, then the discussion section of the ms would have to discuss the reasons behind these discrepancies.

Thus, we would like to only include the highly-standardized and focused road surveys in this ms. Once we have shown that the change in highway lighting did not have an effect, future papers can investigate island-wide fallout patterns using a larger dataset (2012 – 2021).

**Comment 4:** *Personally I would also suggest changing the title, moon and wind effects on fallout are known and not novel, their interaction is expected, on the other hand LEDs effects on fallout are unknown, understudied and needed!*

**Response:** Following this reviewer’s suggestion, we have changed the title of the ms to reflect the influence of changing lighting conditions, rather than the environmental drivers (moon and wind). However, because two changes in lighting conditions took place simultaneously: light bulb (HPS to LED) and light fixture design (unshielded to shielded), we do not explicitly mention LEDs in the title. Thus, our revised title states: “Quantifying wedge-tailed shearwater (*Ardenna pacifica*) fallout after changes in highway lighting on Southeast Oʻahu, Hawaiʻi”

**Comment 5:**  *The authors collected data on utility poles (lighting systems) nearest the grounded bird. They could present an analysis of this data, i.e. was it possible to identify specific areas within the transects or fallout was widespread across it? Does this coincide with the previous research of Friswold et al 2020 (so data from 2002-2010) ? it could be interesting to discuss the implications of, after the change in lighting systems, the locations for fallout remain the same or change, especially in relation to the colonies you identify in line 138-142.*

**Response:** We absolutely agree with this comment! In fact, after investigating the influence of the lighting regimes on overall fallout first, we performed a detailed spatial analysis of these fallout data. We chose to focus this first ms on the lighting regime and will publish the fine-scale spatial analyses on a second ms. We felt that publishing both analyses together would yield a rather long and unfocused ms.

**Comment 6:** *The authors only used data of dead birds. Were live birds observed during the transects and if so were they added to the rescue center tally? I miss some discussion regarding the dead versus live birds in relation to the surveys. I understand that the authors provided a parallel between the two datasets by comparing proportions of live (rescue center data) and dead (this study), but if possible it would be interesting to include the live rescued birds in the full models.* *For instances the change to LED did not provide increased deaths however is it possible to evaluate its effect on the total fallout numbers provided by the rescue center? I understand that it might not be possible to confirm the location of fallout for all birds and that the rescue center possibly obtains birds from outside the survey area, however if it were possible to include the birds that have been rescued within the survey area it would greatly improve the results and further increase the impact of this work. Thus discussion not only the overall negative effect of the light pollution (fallout) and the ‘no-effect’ of the light change as well as the mortality associated with both.*

**Response:** First of all, we would like to clarify that our surveys involve both live and dead birds. Whenever we found live birds along the road, we recorded them for our survey and brought them to Sea Life Park. Yet, the vast majority (99%) of the WTSH we encounter during our road surveys are dead, because by the time our surveys occur in the morning, most of the grounded shearwaters have either made their way off the road or have been hit by cars.

Regarding the suggestion to include the SLP intake records of rescued birds in the analysis, we would like to keep these two distinct datasets apart due to their inherently different spatial / temporal scales. Please see previous explanation in response to a similar suggestion by reviewer #1.

The reasons why we included the SLP intake records in this paper were to: (i) show that our surveys from Nov 6 to Dec 21 sampled the period of fallout throughout the island; and (ii) show that – despite the small geographic scale of our study, the year-to-year variability in fallout we documented was correlated to the pattern of island-wide fallout. Because there was a significant cross-correlation of both datasets (which shared 85% of their year-to-year variability), we contend that the same interannual drivers (e.g., ocean productivity, weather patterns) of island-wide fallout also influence the magnitude of fallout within the hotspot in the SE corner of the island.

**Comment 7:** *Figure 4 is missing. If possible maybe combine the two time series graph (I understand this will generate three y scales but perhaps there is a way to illustrate the moon illumination as well).*

**Response:** Apologies for erroneously leaving out Figure 4. I have added it back in. I had considered combining the two time series, and attempted it, but felt that it made the graph difficult to read and interpret, thus decided to keep them separate.

**Comment 8:** *reduce the size of paragraph 5 in the introduction*

**Response:** We removed some text and condensed the introduction.

**Comment 9:** *methodology could be shortened and the information placed in a supplementary material, for example the passages pertaining to the specificity of Poisson distributions and the AIC (lines 192-195; 211-221)*

**Response:** We removed some text and reorganized this section, which includes a general opening and then splits into two modelling sections (multi-year vs yearly models). This new organization removed some redundancy

**Comment 10:** *If possible include 95% Confidence intervals in the results from your models. I fell it would provide a more cohesive interpretation of the results.*

**Response:** While we see how adding 95% confidence intervals may enhance the interpretation of the results, we included merely the parameters and associated values for two reasons: 1) logistically, we wanted to keep the table small ensure that it was not overwhelming and 2) statistically, because the yearly models are based on small sample sizes (n=16), they provide an exploratory perspective. Thus, we want to de-emphasize the specific parameter values. Rather we only provide the parameter estimates to show the +/- signs and the p-values for reference. If we added the SE’s we would also want to add Z scores which would add two more columns for each explanatory variable. On the other hand, because the multi-year model has a much larger sample size (n=128) this analysis provides a more rigorous statistical analysis. Thus, when we report this model’s result, we provide the parameter values, +/- SEs, Z scores, and p-values so the reader can fully evaluate the significance of the model.

**Minor Changes:**

*>I have added line numbers to the text version of the ms as they were missing.*

Sorry for not including line numbers. I have added them in to my final draft.

*> Careful with the use of abbreviations such as HDOT. PLOS ONE guidelines state ‘Do not use non-standard abbreviations unless they appear at least three times in the text.’*

I removed the abbreviation and spelt out the acronym.

*> in the CCT mentions throughout the text, remove the degree symbol before K (absolute temperature Kelvin is not used with the degree symbol)*

Thank you for making me aware of this. I have removed the degree symbol.

*> line 43. Remove comma after petrels: ‘*petrels *and puffins’*

Done

*> line 50. ‘conditions’ is repeated*

Thank you for catching this mistake.

*> line 60. Add reference regarding powerline collisions Travers et al. 2021 Avian Conservation Ecology*

Thank you for sharing this reference. I have added it.

*> line 79. I disagree that a CCT >2700K is high. I would substitute by ‘recommended’ for example. Organism friendly.*

Thank you for this recommendation. I have changed the wording.

*> line 292 and 293. Revise text. Two sentences are unconnected, ‘yet’ followed by ‘however’ is confusing.* Thank you. Rephrased.

*> Table 1. Correct to ‘mean ratio (VMR).*

Not sure what is meant with this suggestion. The text states” variance to mean ratio”.

*> Figure 3. Perhaps it would be appropriate to add a line plot with total of birds from the rescue centre.*

We respectfully disagree because we have provided the yearly totals in S1 Table, and in Figure 2.

*> Figure 5. I find this particularly useful for the wind and moon integrated evaluation. The smaller circles represent 0 fallout? I would substitute all 0 fallout records with a different symbol, for example an ‘x’ or a ‘+’. As it is its not possible to discern surveys with no fallout or surveys with low fallout. I wonder if using two colors to represent the two light regimes would also be usefull in this graph.*

Thank you for this suggestion. We changed the zero values from an open circle to a small solid black dot, and edited the figure legend to explain the symbology.

**Comments from Reviewer 3**

**Comment 1:** *Page 4, last paragraph: “wedge-tailed shearwaters” should be capitalized.*

**Response:** Thanks for catching that! Capitalized.

**Comment 2:** *Page 5, second line: USFWS should be spelled out the first time this acronym is used.*

**Response:** Good point. Spelled out.

**Comment 3:** *Page 6, second paragraph, last sentence: The prediction that northeasterly winds would lead to higher fallout rates goes against the observation that more birds were found stranded during anomalous southerly winds in 1994, as stated on page 5, second paragraph. Please clarify. Also, it would be useful to add an explanation of why >8 mph winds was chosen as “strong”. For example, in other parts of the world, winds under 13 mph would be classified as low, so it would be good for the reader to have a better understanding of what typical wind speeds are for this particular region. Also, for consistency, it would be better to present all wind speeds as knots, rather than a combination of mph and knots.*

**Response:** We have reworded our expectations by removing the 8 knot threshold and by explaining the link between NE winds and shearwater deposition along the SE shore of the island, downwind from two nesting colonies on offshore islets. Now, the text states: “Because WTSH rely on wind to take flight and may become disoriented in the absence of moonlight, we predicted higher fallout during windy nights of low moon illumination. In particular, due to the location of our study area, southwest from two breeding colonies, we anticipated that strong northeasterly winds would drive the fledging birds towards shore”. We provide an explanation below and can include similar wording in the ms, if you deem that it would help clarify our hypothesis.

When relating WTSH fallout to wind direction, we expect different patterns when we compare the counts of grounded birds within our small-scale study area versus the island-wide results. Work and Rameyer (1999) analyzed island-wide patterns in 1992 – 1994, using Sea Life Park (SLP) intake records. They reported widespread fallout in 1994, with WTSH distributed throughout the windward (east), leeward (south and west) and north shores of the island (see enclosed figure).

Yet, while WTSH fallout was widely distributed in 1994, when southerly winds deposited the birds throughout the entire island, large numbers of grounded birds were found within our study area, in the vicinity of the breeding sites on offshore islets (in the SE corner of the island). This result underscores the correlation we documented between SLP intake records and our road surveys.

Thus, we hypothesize that strong winds lead to higher fallout. In particular, we feel like the tradewinds (NE winds) will deposit birds down-wind from the offshore islet colonies, leading to highly focused fallout in our study area, rather than widely-dispersed fallout throughout the island.

**Comment 4:** *Table 1: the p-values for moon and date were cut-off from the table in the produced document.*

**Response:** We apologize if the table was not readable in this format. The journal submission guidelines stated not to reformat the table if it did not fit within the page dimensions, because it will be turned sideways and displayed in landscape orientation.

**Comment 5:** *While the Figure 4 caption is present in the text, the actual figure is missing from the document.*

**Response:** Apologies for erroneously leaving out Figure 4. We added it back in.

**Additional Editorial Comments:**

- Update formatting and file name - upload figures as individual files (Fig1.tif)

Done

- Add supporting information at the end of manuscript after refs

Done

- 5. Please include a copy of Table A1 which you refer to in your text on page 11.

We removed Table A1, which originally referred to the table showing all tested model combos. Now, Table A1 refers to the fallout summary from the SLP Intake Records and the Road Surveys

- 6. Please upload a copy of Supporting Information Figure S1 and S2 which you refer to in your text on page 18 and 19

We added these figures to the supplementary materials

- Update the role of the funder

We updated the role of the funder

- Make data publicly available

We provide a copy of the data used for the fallout models as S Table 2

- Make sure copyright info is all good for figure 1 map - added line in caption indicating that map was made in ArcGIS Pro and added ArcGIS Pro reference in references section. I made this map, it was no taken from another source.
