# Supplementary material for: Quantifying wedge-tailed shearwater (Ardenna pacifica) fallout after changes in highway lighting on Southeast Oʻahu, Hawaiʻi
Source: PLoS One. 2022 Mar 24;17(3):e0265832. doi: 10.1371/journal.pone.0265832 (PMC8947263; doi:10.1371/journal.pone.0265832)
Supplement: Attachment — Submitted filename: PONE-D-21-31078_R2 Response to Reviewers_0306_2022.docx [file pone.0265832.s008.docx]

**Response to Reviewers**

Re: Response to Reviewers (PONE-D-21-31078)	03/06/2022

Dear Dr. Vitor Hugo Rodrigues Paiva,

Thank you for giving us the opportunity to submit a second revised draft of our manuscript entitled “Quantifying wedge-tailed shearwater (*Ardenna pacifica*) fallout after changes in highway lighting on Southeast Oʻahu, Hawaiʻi”. We sincerely appreciate the level of detail that you and the reviewers have put into improving our manuscript. We have incorporated all suggested edits and have addressed all reviewers’ comments. Below we provide a point-by-point response to each of the comments and suggestions made by reviewers.

We are forwarding two versions of the revised (R2) ms: (i) a “marked-up” version includes all the edits we implemented using track changes in Microsoft Word, and (ii) a “clean” version after accepting all these changes and edits. Below, we also provide a point-by-point response to the reviewers’ comments and concerns.

We look forward to your decision.

Sincerely,

Jennifer Urmston, Keith Swindle and David Hyrenbach

**Editor**

Comment: Please review your reference list to ensure that it is complete and correct. If you have cited papers that have been retracted, please include the rationale for doing so in the manuscript text, or remove these references and replace them with relevant current references. Any changes to the reference list should be mentioned in the rebuttal letter that accompanies your revised manuscript. If you need to cite a retracted article, indicate the article’s retracted status in the References list and also include a citation and full reference for the retraction notice.

Response: After double-checking the references, I did not find any articles that have been retracted. The only reference that is no longer available is the US Naval Observatory Moon Phase data. This web-site was available during the duration of my study, but the link is no longer active. However, historical moon phase data can be found at timeanddate.com which match the data I retrieved from the US Naval Observatory.

**Reviewer 1**

Comment #1 Line 40 – remove unnecessary bracket.

Response: Thank you for catching this mistake, I removed it.

Comment #2 Line 42 – USFWS should be spell out.

Response: Thank you for this suggestion, I have spelled it out.

Comment #3 Table 1 – spell out Wind Speed, as WS is not clear.

Response: Thank you for this suggestion. I have typed out wind speed in table #1. I left the abbreviation in table 2 as it is identified in the figure caption, and there is limited space in the column headings.

Comment #4 Line 275 – I am uncertain what do you mean by ‘larger degree angle’? Maybe try wind coming from land/offshore.

Response: Thank you for your comment. I agree that ‘larger degree angle’ is not very clear. I have changed it to say “wind blowing from the southwest” which I believe will be more intuitive to the readers.

Comment #5 Line 289 – can you specify ‘a single new moon period’

Response: Changed to “a new moon period occurred in the middle of the fledging season, leading to a single peak in fallout”. This is opposed to the previous line describing a year with two new moon periods, early and late in the season.

Comment #6 Lines 406-408 – It could be also a result of other moon cycle variables (timing, location of moon in respect to earth) and environmental conditions (cloud cover) that were not taken into account in the model.

Response: If the periodic moon cycle or other episodic environmental drivers (cloud cover and wind) were the drivers of fallout, we would expect that the 9-year (2002 – 2010) dataset of daily fallout records to yield a broad fallout distribution, caused by the overlapping of distinct annual peaks with different timing. Instead, we hypothesize that the consistent weekly fallout peak (Nov. 21 – 27) is caused by the timing of fledging, riven by the phenology of chick hatching and development. Nonetheless, I inserted more detail in this section:

“When we replicated this analysis for WTSH, the quadratic model was not significant (R^2^=0.038, F_2,5_=1.140, p=0.39), suggesting that annual fallout did not follow the same pattern with the timing of the full moon. Although, other variables such as the timing of moon rise, cloud cover, and topography blocking the moon were not taken into account and may play a role in the moon’s influence on fallout.”

Comment #7 Figure 4 – I do not see solid black dots indicating absence of fallout.

Response: Thank you for catching this. I changed it to “small solid dots”, they are grey not black. I have updated the figure caption.

**Reviewer 2**

Comment #1

Still unclear in the text regarding the state of collected birds: I understand from the authors that both live and dead birds were collected during the surveys, even if 99% of birds were dead. I maintain my recommendation to include this information in the ms, thus facilitate comparison with other studies and clarifying the work. For example: in methods explain that both live and dead birds were collected but since most were found dead (99%) only these were used for the analysis (unless all were used for the analysis, in which case please correct line 127 and other mentions of 'carcass' across the text); In the discussion, mention that even if this study only used dead birds, as you have found a good parallel between SLP intakes (island wide records of fallout, both live and dead) and this study, there is no evidence for a different effect of light change to the state of the birds, for example. These additions do not need to be lengthy, a short sentence will suffice.

Response: Thank you for these suggestions. I have added some clarifying language to distinguish that our surveys focused on dead birds, and we only used dead birds in our analysis.

“while visually searching for dead birds in each lane and along the shoulder. Since these surveys were conducted in the morning, likely a full 12 hours after fledging time, almost all the birds we observed were deceased. In 8 years of surveys, we observed 2 live birds, which were brought to Sea Life Park for rehabilitation and not counted in our analysis. All dead birds sighted”

“Our results are reassuring because they suggest that the shielded LED streetlights did not increase WTSH mortality due to fallout, as we hypothesized. Given the strong correlation between the dead birds observed in our road surveys and the live birds brought to SLP, there is no evidence suggesting that the shielded LED streetlights impacted the number of birds affected by fallout overall.”

Comment #2

Introduction: 4th paragraph (line 31-38) is a bit redundant, it could be shortened to be more concise and direct.

Response: Thank you for addressing this. I agree that this paragraph sounded misleading. I have rephrased this section to address the point that many newly implemented lights feature both shielding (good for birds) AND broad spectrum LEDs with high CCT values (potentially bad for birds). So the point I am trying to make is that we have a good change and a potentially bad change happening at the same time, and we don’t know how these changes coupled together are impacting birds.

“Mitigation measures often target light directionality, whereby streetlights are shielded through the use of a “full-cutoff” design, which inhibits light emission above the horizontal plane of the fixture. This approach, when applied to HPS lights, reduced Newell’s Shearwater (*Puffinus newelli*) fallout on Kauai (Hawaiʻi) [16]. Although mitigation is being addressed through shielding, the common use of optimized LEDs with broad spectra and Correlated Color Temperature (CCT) greater than the maximum recommended value for wildlife (2200 K) may be a cause for concern [17]. While modern LED lights possess the flexibility to give off a range of low to high CTT, short-wavelength light with high CCT is a common choice because of its efficiency [19]. The effectiveness of light shielding coupled with the use of broad spectrum, high CCT LEDs is unknown.”

Comment #3

Abstract: 'due to exhaustion or collision' (use singular for collision).

Response: Thank you for the suggestion, I have made this edit.

Comment #4

Line 14 - missing word: 'especially in the absence of "moonlight"...'

Response: Thank you for your thorough review and for catching this mistake! I have added in the word.

Comment #5

Line 20 - 'that can affect seabirds' or better yet, 'that can affect fallout'?

Response: Thanks for the suggestion, I have changed the phrasing to say “that can affect fallout”.

Comment #6

line 64: 3000-4000 K LED lights (missing LED)

Response: Thank you, I have added in “LED”

Comment #7

Line 64: I think (might be wrong) that is more accurate to say that CCT is the measure of how warm and cold a light appears, Kelvin is the unit.

Response: I think you are correct. I have rephrased this to say “where Kelvin (K) is a unit of measurement for CCT. Lower CCT indicates a warm yellow-orange appearance whereas higher CCT indicates cool blue light [18].”

Comment #8

Line 123: I would use full extent month names throughout the text (November 6 instead of Nov.). Or at least homogenize across the text.

Response: Thank you for pointing this out. I used full names in text and left abbreviations in tables only.

Comment #9

Line 285: instead of 'throughout this study' something like 'within the time frame of this study'

Response: Good suggestion, made change.

Comment #10

Line 324: distinguish between these two factors

Response: Thanks for the suggestion, I have made this edit.

Comment #11

Line 337: I agree that evidence indicates stronger winds will increase number of fledgling. But wind affects groundings two ways a) increase the number of fledglings and b) push these inland (especially when southwestern winds are about correct?). I would add this last part. you can reference 10 and 11 refs [Rodríguez et al 2014 and Syposz et al 2018]

Response: I agree that this is likely the case, but this section is specifically referencing our multi-year model, which identified the Wind Speed * Moon interaction as the single most important variable, but did not include wind direction. Thus, we would like to avoid referring to wind speed and the advection of chicks inland. We are developing a second ms analyzing the spatial pattern of fallout in relation to wind speed and direction, and this ms will analyze and discuss those patterns.

**Reviewer 3**

Comment #1

Reviewer #3: While the authors have addressed the previous round of comments, I have noticed some conflicting information in the introduction which I feel needs to be resolved as it impacts the logic of the main hypothesis. Line 34 states that light with high CCT (>2700K) is recommended for wildlife (note that a reference to support this statement is needed). Line 64 states that streetlights were changed from 2200K HPS lights to shielded 3000-4000 K LED lights (note that the word "LED" is missing in line 64 and should be added). Following the logic from the former statement (i.e., that light with >2700K is recommended for wildlife), the new LED light regime would be beneficial to shearwaters, however, the hypothesis states the opposite. Furthermore, the hypothesis is based on differences in wavelength rather than on CCT and K which are the light characteristics (related to temperature) discussed in detail in the introduction. Pulling all this together, it appears that the main hypothesis is based on the previous study of shearwaters which showed maximum light absorption by white LED lights emitting short wavelengths vs HPS lights which emit longer wavelengths, which presumably means that they are more attracted to white LED lights. As currently written, the introduction lacks a clear description of the relationship between the two light characteristics presented, namely: temperature and wavelength, and how these informed the predictions of the hypothesis.

Response: Thank you for these comments. I agree that this section sounded misleading. I have rephrased this section to address the point that many newly implemented lights feature both shielding (good for birds) AND broad spectrum LEDs with high CCT values (potentially bad for birds). The point I am trying to make is that we have a good change and a potentially bad change happening at the same time, and we don’t know how these changes coupled together impacts birds.

Also thank you for stating that a description of the relationship between wavelength and CCT is lacking, I have added this in the introduction:

“Mitigation measures often target light directionality, whereby streetlights are shielded through the use of a “full-cutoff” design, which inhibits light emission above the horizontal plane of the fixture. This approach, when applied to HPS lights, reduced Newell’s Shearwater (*Puffinus newelli*) fallout on Kauai (Hawaiʻi) [16]. Although[JU1] [JU2] mitigation is being addressed through shielding, the common use of optimized LEDs with broad spectra and Correlated Color Temperature (CCT) greater than the maximum recommended value for wildlife (2200 K) may be a cause for concern [17]. While modern LED lights possess the flexibility to give off a range of low to high CTT, short-wavelength light with high CCT is a common choice because of its efficiency [19]. The effectiveness of light shielding coupled with the use of broad spectrum, high CCT LEDs is unknown.”

Additionally I corrected this line and provided a proper reference:

Corrected this line - added reference: greater than the recommended value for wildlife (<2200 K) may be a cause for concern [17].

Other minor edits:

Comment #2

Line 20: "seabird" should be plural

Response: I changed this to say “fallout” instead, as per Reviewer 2 recommendation

Comment #3

The sentence in lines 65 and 66 seems out of place as it talks about CCT while the previous sentence talks about K; the latter sentence needs to be tied somehow to the previous one.

Response: Thank you - rephrased 3000 – 4000 K LED lights, where Kelvin (K) is a unit of measurement for CCT. Lower CCT indicates a warm yellow-orange appearance whereas higher CCT indicates cool blue light [18].
